# Interleukin-6 Is Crucial for Recall of Influenza-Specific Memory CD4^+^ T Cells

**DOI:** 10.1371/journal.ppat.1000006

**Published:** 2008-02-29

**Authors:** Maria Paula Longhi, Kate Wright, Sarah N. Lauder, Mari A. Nowell, Gareth W. Jones, Andrew J. Godkin, Simon A. Jones, Awen M. Gallimore

**Affiliations:** Medical Biochemistry and Immunology, School of Medicine, Cardiff University, Heath Park, Cardiff, United Kingdom; Mount Sinai School of Medicine, United States of America

## Abstract

Currently, our understanding of mechanisms underlying cell-mediated immunity and particularly of mechanisms that promote robust T cell memory to respiratory viruses is incomplete. Interleukin (IL)-6 has recently re-emerged as an important regulator of T cell proliferation and survival. Since IL-6 is abundant following infection with influenza virus, we analyzed virus-specific T cell activity in both wild type and IL-6 deficient mice. Studies outlined herein highlight a novel role for IL-6 in the development of T cell memory to influenza virus. Specifically, we find that CD4^+^ but not CD8^+^ T cell memory is critically dependent upon IL-6. This effect of IL-6 includes its ability to suppress CD4^+^CD25^+^ regulatory T cells (Treg). We demonstrate that influenza-induced IL-6 limits the activity of virus-specific Tregs, thereby facilitating the activity of virus-specific memory CD4^+^ T cells. These experiments reveal a critical role for IL-6 in ensuring, within the timeframe of an acute infection with a cytopathic virus, that antigen-specific Tregs have no opportunity to down-modulate the immune response, thereby favoring pathogen clearance and survival of the host.

## Author Summary

Influenza virus poses a serious global health threat, particularly in light of newly emerging strains such as the avian virus H5N1. The generation of cell-mediated vaccines against influenza virus requires an understanding of mechanisms underlying effective virus-specific T cell memory. This study explored the impact of a cytokine, interleukin-6 (IL-6), on generation of effective influenza-specific T cell memory. This cytokine was considered important based on previous studies revealing its role in promoting survival and activity of conventional T cells whilst inhibiting the activity of T cells involved in dampening down immunity (regulatory T cells). We found that the activity of a subset of influenza-specific memory T cells (CD4^+^ T cells) was diminished in the absence of IL-6 due to the inhibitory effects of regulatory T cells—an effect that compromised protective anti-viral immunity. Since a robust CD4^+^ T cell response is likely to be central to the success of a vaccine against influenza virus, these findings highlight the importance of IL-6 in promoting effective cell-mediated immune responses, thereby facilitating successful virus clearance.

## Introduction

Infection with influenza virus is associated with significant mortality particularly amongst children and the elderly. The emergence of new strains, most notably avian virus H5N1, poses an increasing pandemic threat underlining the need for further studies into generation of anti-viral immunity. A consideration of how cytokines modulate cellular immune responses will facilitate a better understanding of the interplay between such pathogens and the immune system and provide rationale for enhancing vaccine efficacy. Interleukin (IL)-6, a multifunctional cytokine expressed by both lymphoid and non-lymphoid cells (reviewed in [1]), has been implicated in increasing the severity of disease in humans infected with influenza virus, including H5N1. However, murine studies indicate that IL-6 does not contribute significantly to the pathogenesis of influenza virus, since the rate of morbidity and mortality observed in mice infected with H5N1 are comparable in both wild-type and IL-6 deficient mice [2],[3]. IL-6 does however play a pivotal role in regulating the immune system – the cytokine plays a central role in resolving the innate immune response and directing the transition from innate to acquired immunity – a process that can, at least in part, be attributed to its effect on recruitment, activation and survival of different leukocyte subsets (reviewed in [4]). Although IL-6 is classically defined as a central regulator of the acute phase response, it is increasingly evident that IL-6 performs a pivotal role in influencing T cell responses. This role of IL-6 appears to be crucial for the progression of autoimmune conditions such as rheumatoid arthritis and Crohn's disease where IL-6 has been implicated in the retention of activated T-cells within the affected tissue, a finding that might provide a mechanistic basis for the highly therapeutic action of blocking IL-6R antibodies in these diseases [5],[6]. However IL-6 control of this process may represent only part of the story, since IL-6 also plays a role in orchestrating T-cell polarization, proliferation, survival, and effector function (reviewed in [4]). Collectively, these studies point to a critical role for IL-6 in directing antigen-specific T cell responses. To date however, the influence of IL-6 on development of antigen-specific T cell memory has not been explored. In this study we compared influenza-specific CD4^+^ and CD8^+^ T cell memory after infection of wild-type (WT) and IL-6 deficient (IL-6^−/−^) C57BL/6 (B6) mice with influenza virus. The results of this study were striking and defined IL-6 as a selective regulator of long-term CD4^+^ T-cell memory responses, through its capacity to limit the activity of virus-specific Tregs.

## Results

### Influenza-specific CD4^+^ T cell responses but not CD8^+^ T cell responses are impaired in IL-6^−/−^ mice

**Figure 1:**
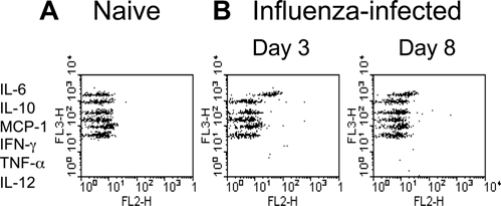
IL-6 production post-infection with influenza virus. WT mice were infected i.n. with 20 HAU H17 influenza virus. Three and eight days after infection, serum levels of IL-6, IL-10, MCP-1/CCL2, IFN-γ, TNF-α, and IL-12 were measured in uninfected (A) and infected (B) mice by Cytometric Bead Array.

We assessed whether IL-6 is produced following infection of mice with influenza virus. Serum, removed from WT mice infected intranasally 3 and 8 days earlier with 20 haemagglutination units (HAU) of influenza virus, was assessed by Cytometric Bead Array. This method of analysis allowed us to compare serum levels of IL-6, IL-10, MCP-1, IFNγ, TNFα and IL-12 in the infected mice. The results, shown in Figure 1, indicate a selective elevation in IL-6 secretion in the serum at these time-points. IL-6 levels returned to baseline levels following the establishment of the memory phase of the infection (data not shown). Since IL-6 production was prominent during the acute phase of infection and within the timeframe of virus specific T cell activation we considered it reasonable to hypothesize that IL-6 plays a role in shaping the T cell response to influenza virus.

### Influenza-specific memory CD8^+^ T cell responses were unimpaired in IL-6^−/−^ mice

**Figure 2:**
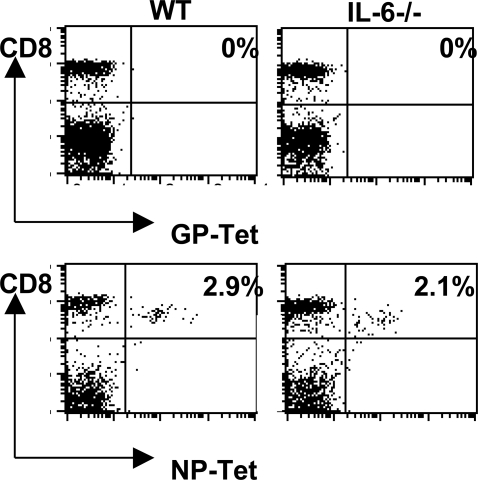
Nucleoprotein (NP)-Tetramer-positive CD8^+^ T cells in the spleens of influenza virus-infected mice. Representative dot plots showing staining of spleen cells recovered from a WT and IL-6−/− mouse 10 days post i.n. infection with 20 HAU H17 influenza virus. The cells were stained with CD8-specific mAbs and either the NP-Tetramer (Db-ASNENMDAM, NP-Tet) or an irrelevant tetramer comprising D^b^ and a peptide derived from Lymphocytic choriomeningitis virus (D^b^-KAVYNFATC, GP-Tet). The plots are representative of 3 mice per group.

**Figure 3:**
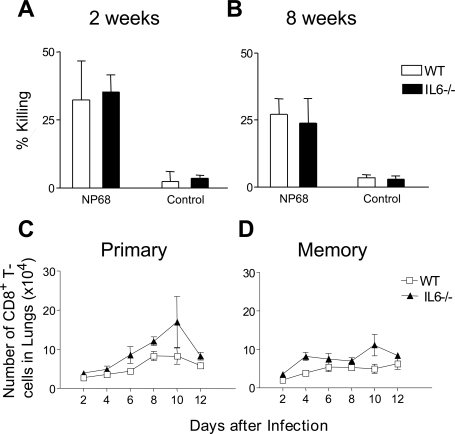
Influenza specific CD8^+^ T cell activity in WT and IL-6^−/−^ mice. Peptide-specific CTL assays were carried out at 2 weeks (A) and 8 weeks (B) post i.n. infection with H17 influenza virus. B16 target cells were pulsed with the NP68 peptide prior to use in a standard 5-h ^51^Cr release assay. Total number of CD8^+^T cells recovered from lungs of WT and IL-6−/− mice following primary (C) or secondary (D) i.n. challenge with H17 influenza virus. Mice were analyzed individually, and values shown are the mean±SD (*n* = 3 mice/group). The results are representative of three independent experiments. Statistical significance was evaluated using the Student's *t* test.

To define the role IL-6 performs in orchestrating the immune response to viral infection, *ex vivo* studies tested whether IL-6 could affect primary and memory T cell responses to influenza infection. Firstly, CD8^+^ T cell activity was compared using cells derived from WT and IL6^−/−^ mice following influenza virus infection. MHC class I tetramers comprising the D^b^-ASNENMDAM complex were used to stain lymphocytes recovered during the primary phase of the infection (day 10) and 8 weeks post-infection. The data shown in Figure 2 indicates that no significant difference was observed between the groups at day 10 post-infection whilst no tetramer positive cells were observed in either group following *ex vivo* staining of spleen cells 8 weeks post-infection (data not shown). Functional activity of these cells was assessed by CTL assay (Figure 3A and B) and again, no difference was observed in influenza-specific cytotoxic activity measured in spleen cells isolated from WT and IL-6^−/−^ mice 2 and 8 weeks post-infection. The presence of CD8^+^ T cells in the lungs of both WT and IL-6^−/−^ mice was also measured during days 2–12 of a primary (Figure 3C) and secondary infection with influenza virus (Figure 3D). A similar CD8^+^ T cell response was observed in the lungs of WT and IL-6^−/−^ mice during the primary infection and following rechallenge with the same virus. Overall, these data indicate that lack of IL-6 had no significant effect on the generation or recall of the CD8^+^ T cell response to influenza virus.

### Influenza-specific memory CD4^+^ T cell responses are impaired in IL-6^−/−^ mice

**Figure 4:**
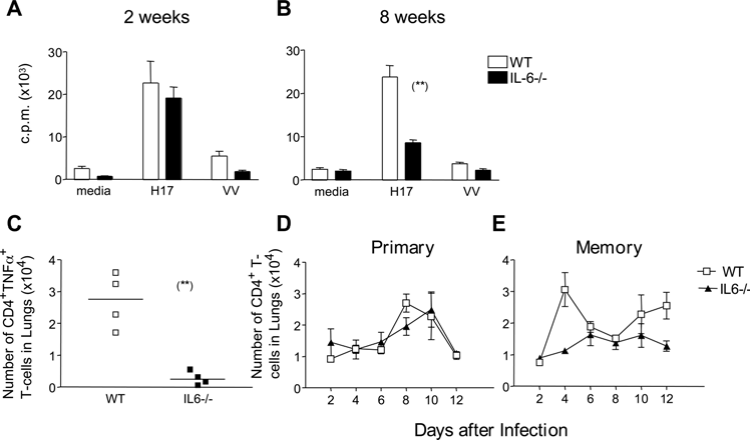
Influenza specific CD4^+^ T cell activity in WT and IL-6^−/−^ mice. Proliferation assays were carried out using CD4^+^ T cells purified from spleens of WT and IL-6^−/−^ mice 2 weeks (A) and 8 weeks (B) post i.n. infection with 20 HAU H17 influenza virus. Effectors were incubated with irradiated WT splenocytes alone or WT splenocytes infected with inactivated H17 influenza or vaccinia virus. [^3^H]-Thymidine was added on day 5 and proliferation measured by thymidine incorporation after 18 hrs. Mice were analyzed individually and values shown are the mean±SEM (n = 3 mice/group). Spleen cells were isolated 8 weeks after primary H17 infection and influenza-specific CD4^+^ T cells were analyzed for intracellular TNF-α by flow cytometry (C). Each symbol represents an individual mouse. Kinetic analysis of total CD4^+^ T cells infiltrated in lungs from WT and IL6−/− mice following primary (D) and secondary (E) i.n. challenge with 20 HAU H17 influenza virus. Mice were analyzed individually and values shown are the mean±SD (n = 3 mice/group). Statistical significance was evaluated using the Students *t* test.

In light of the ability of IL-6^−/−^ mice to generate a comparable CD8+ T cell response to WT mice, influenza-specific CD4^+^ T cell responses were also assessed in both groups of mice. Virus-specific proliferation assays using ^3^H-thymidine incorporation were performed on WT and IL6^−/−^ splenic CD4^+^ T cells, isolated either 2 weeks post infection (primary CD4^+^ T cell response), or at 8 weeks following a secondary infection (memory CD4^+^ T cell response). At 2 weeks post infection no difference was observed in influenza-specific CD4^+^ T cell proliferation between WT and IL6^−/−^ mice, with both groups exhibiting robust virus-specific responses (Figure 4A). However, at 8 weeks post infection, the difference between the two mouse strains was striking. Whilst virus-specific proliferation was readily observed in WT mice, a notable reduction in proliferation was observed in IL6^−/−^ mice (Figure 4B), indicating that influenza-specific memory CD4^+^ T cell responses are severely impaired in the absence of IL-6. In further support of this, we found that whilst TNFα-producing CD4^+^ T cells could be observed following a 5 hour *in vitro* restimulation of CD4^+^ T cells isolated from the spleens of WT mice infected 8 weeks previously with influenza virus, these cells were not observed following restimulation of CD4^+^ T cells purified from IL-6^−/−^ mice (Figure 4C). Collectively these data imply that the activity of influenza-specific central memory T-cells (T_CM_ cells, which retain proliferative capacity after initial antigen challenge) and effector memory T cells (T_EM_, which produce cytokines rapidly upon re-exposure to antigen) are both impaired in IL-6^−/−^ mice. To extend these observations, we assessed CD4^+^ T cell responses *ex vivo* by measuring the presence of CD4^+^ T cells in the lungs of both WT and IL-6^−/−^ mice following the first and second exposure to influenza virus. For this purpose, WT and IL6^−/−^ mice were initially infected (i.n) with influenza virus and then subsequently re-infected by the same route 8 weeks later. CD4^+ ^T cell numbers in the lungs were then recorded during the first 2–12 days of the first (Figure 4D) and second infection (Figure 4E). Although both WT and IL-6^−/−^ mice elicited primary responses to influenza infection, the overall profile of CD4^+^ T cell infiltrating of the lung indicates that the T cell response was slightly delayed in IL-6^−/−^ mice consistent with previous reports (Figure 4D) [7]. These differences were however more pronounced following the second viral challenge (Figure 4E). In this respect, the number of CD4^+^ T cells in the lungs was dramatically reduced in IL-6^−/−^ mice as compared to WT mice. Whilst a defect in trafficking may contribute to this reduction in T cell number, the results are also consistent with a profound failure of the influenza-specific CD4^+^ T cell memory response detailed in Figures 4B and C above.

**Figure 5:**
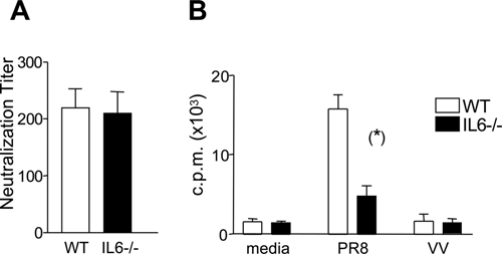
Neutralizing activity in the serum of the H17 influenza virus memory mice. (A) Serum from WT and IL-6^−/−^ mice was harvested at 6–8 weeks post i.n. infection with 20 HAU of H17. MDCK cells and serum samples were cultured in the presence of 10 HAU of H17 and the viability of the cells determined 3 days later using the alamar blue assay. The presence of neutralizing antibodies in serum was determined by comparing cell viability of MDCKs cultured with serum from naïve mice, with viability of MDCKs cultured with serum from previously infected mice (see Materials and Methods for calculation). Results are presented as the mean neutralization index score±SEM at each dilution (n = 6–7 mice per group). (B) T cell cross-reactivity of PR8 and H17 was measured in a CD4^+^ T cell proliferation assay. Mice were infected i.n. with 20 HAU H17 virus and 8 weeks later CD4^+^ T cells were purified and stimulated *in vitro* with PR8-infected APCs. Proliferation was measured by [^3^H]-thymidine incorporation at day 5. Each bar represents the mean value±SEM (n = 3 wells/group). Statistical significance was evaluated using the Students *t* test.

**Figure 6:**
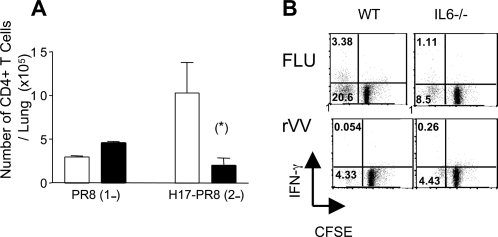
Challenge of H17 influenza virus immune mice with the heterologous virus, PR8. WT and IL-6^−/−^ mice infected 6 weeks previously with H17 influenza virus were rechallenged i.n. with 20 HAU of the PR8 virus. Four days post secondary infection the lungs were harvested and the number of CD4^+^ T cells analyzed by flow cytometry (A). Proliferation and cytokine production was measured in splenocytes of H17-immune mice re-challenged 4 days previously with PR8 virus (B). The cells were labelled with CFSE and incubated for 6 days with H17 influenza-infected APCs as described in materials and methods. Specific proliferation was measured CFSE dilution and IFNγ production on gated CD4^+^ cells. The dot plots show a representative of 5 mice per group.

Re-challenge with homologous virus as described above poses the problem that neutralizing antibodies, induced following the primary infection may differ between WT and IL-6^−/−^ mice altering their ability to control the second infection thereby impacting on the extent of the T cell response induced following the second infection. Comparable neutralizing antibody activity was observed in WT and IL-6^−/−^ serum analyzed 8 weeks post infection thus the impairment in the CD4^+^ T cell memory response observed in the IL-6^−/−^ mice did not impinge on their ability to generate adequate neutralizing antibodies (Figure 5A). Since the presence of neutralizing antibodies would confer resistance to re-infection with a homologous virus resulting in very little restimulation of memory T cells, a more stringent test of T cell memory was performed. Mice infected 8 weeks previously with the H17 strain of influenza were rechallenged with the heterologous virus, PR8. This virus was considered appropriate since serum from H17 infected mice failed to neutralize PR8 (data not shown), but CD4^+^ T cells purified from the spleens of WT H17-infected memory mice proliferated *in vitro* when co-cultured with PR8-infected APCs indicating that the viruses share T cell epitopes (Figure 5B). Four days post-infection with the PR8 virus, CD4^+^ T cells in the lungs of both H17-memory and naïve WT and IL-6^−/−^ mice were enumerated by flow cytometry. A pronounced difference was observed in the number of CD4^+^ T cells in the lungs of WT and IL6^−/−^ H17 memory mice following rechallenge with PR8 (Figure 6A). A significant increase in the number of CD4^+^ T cells was detected in the lungs of WT H17-memory mice 4 days after PR8 infection compared to naïve mice infected 4 days previously with the PR8 virus. No such difference was observed in the lungs of similarly infected IL-6^−/−^ mice indicating an impaired recall response in these animals. A comparison of virus burden in both groups of animals revealed higher levels of virus in the lungs of IL6^−/−^ mice compared to the WT mice (Table 1). This is likely to be due to the impaired CD4^+^ T cell responses since no difference was observed in the CD8^+^ T cell response (measured by enumerating tetramer-positive cells in lungs and spleen (data not shown)) induced following rechallenge of both groups of mice with PR8. To confirm that influenza-specific CD4^+^ T cells were functionally impaired in H17-memory mice lacking IL-6, the ability of the cells to proliferate in response to the H17 influenza virus was compared by CFSE dilution (Figure 6B). Spleen cells from both groups of mice were labeled with CFSE and stimulated with APCs infected either with the H17 influenza virus or a control recombinant vaccinia virus for 6 days. The percentage of cells that had proliferated was assessed by CFSE dilution and their capacity to produce IFNγ was assessed by intracellular cytokine staining. As indicated in Figure 6B, more influenza-specific proliferation was observed in the cultures from WT mice than IL-6^−/−^ mice and a higher percentage of these cells produced IFNγ. Collectively, these data confirm that IL-6 plays an essential role in the CD4^+^ T cell memory response to influenza virus. The impact of IL-6 on the influenza-specific T cell response appears to be selective for CD4^+^ T-cells implying that IL-6 has a different effect on the behavior of CD4^+^ and CD8^+ ^T cells. CD4^+^ and CD8^+^ T-cells purified from naïve WT mice express the membrane-bound IL-6 receptor (IL-6R), whilst its expression is downregulated on both populations upon activation ([8] and data not shown). It is not possible therefore to attribute the effect of IL-6 on CD4^+^ and CD8^+^ memory T cells to an obvious difference in classical IL-6 signaling capacity.

**Table 1:** Relative Virus Loads in WT and IL6^−/−^ Mice.

Dilution of Lung Homogenate	Infection of MDCK Cells	Infection of MDCK Cells
	WT Lung	IL6^−/− ^Lung
1/3	+	+
1/9	+	+
1/27	−	+
1/81	−	+
1/243	−	+

Lung homogenates were prepared from mice (n = 4 per group) infected with H17 and rechallenged with PR8 at day 4 post-secondary infection and three-fold dilutions were incubated with MDCK cells. After 3 days cell viability was assessed using the Alamar Blue assay. + denotes dilutions of lung homogenate resulting in infection of MDCK cells. − denotes dilutions of lung homogenate where no infection of MDCK cells was observed.

### Influenza-specific CD4^+^CD25^+^ regulatory T cells inhibit the CD4^+^ T cell memory response in IL6^−/−^ mice

**Figure 7:**
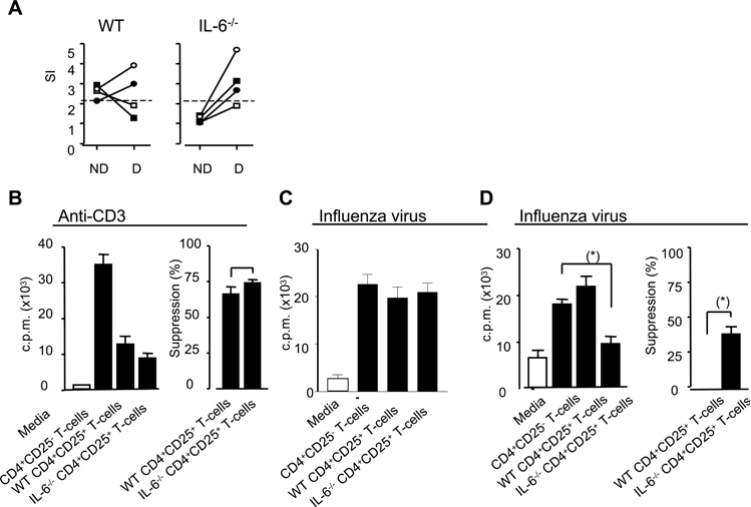
Influenza-specific suppressor activity in WT and IL-6^−/−^ mice. Mice were infected i.n. with 20 HAU of influenza virus (H17). Eight weeks after infection, splenocytes from WT and IL6−/− mice were harvested. (A) Purified CD4^+^ T cells were tested for proliferation against inactivated H17 before (ND) or after (D) depletion of CD25^+^ T cells. Influenza-specific proliferation in both populations was measured by [^3^H]-thymidine incorporation at day 5. Each symbol represents an individual mouse and the lines join responses in undepleted (ND) and CD25-depleted (D) CD4^+^ T cell populations. A stimulation index greater than 2 was considered a positive response. (B) CD4^+^CD25^+^ cells from WT and IL-6^−/−^ mice, infected 2 or 8 weeks previously, were isolated from splenocytes and their suppressive capacity was evaluated by incubation at a ratio of 1∶1 with CD4^+^ T cells from a WT mouse infected 2 weeks previously with H17 influenza virus. The cells were stimulated with either APCs exposed to 1 µg/ml anti-CD3 mAbs (B) or influenza infected APCs (C and D). Proliferation was measured by [^3^H]-thymidine incorporation at day 5. CD4^+^CD25^+^ cells from 4 individual WT and IL-6^−/−^mice were analyzed and each bar represents the mean value±SEM of each group. Statistical significance was evaluated using the Students *t* test.

Since IL-6 has been shown to abrogate suppression by Tregs [9] and to suppress Treg development by TGFβ [10], we postulated that influenza-virus Treg activity would be more readily detectable in IL-6^−/−^ mice than WT mice and that these cells would suppress the activity of virus-specific memory T cells. To test this hypothesis, we first carried out proliferation assays using purified CD4^+^ T cells depleted of CD25^+^ cells from both WT and IL-6^−/−^ mice. In this respect, depletion of CD25^+^ cells had a variable affect on proliferative responses observed in WT mice, removal of CD25^+^ cells uncovered influenza-specific memory CD4^+^ T cell responses in the majority of IL-6^−/−^ mice (Figure 7A). This experiment revealed that influenza-specific proliferative responses can be detected in IL-6^−/−^ mice and suggest that these responses, and to a lesser extent, responses in WT mice, are subject to suppression by Tregs. To more closely examine the responsiveness of Tregs in IL-6^−/−^ mice, we performed suppression assays designed to compare 1) Treg activity following polyclonal stimulation of the cells using CD3-specific mAbs (Figure 7B) and 2) influenza-specific Treg activity (Figures 7C and D) in both groups of mice. Suppression assays were carried out using CD4^+^CD25^+^ Tregs purified from WT and IL-6^−/−^ mice infected 2 (Figure 7C) and 8 weeks (Figure 7D) previously with influenza virus. CD4^+^CD25^+^ T cells derived from these mice were incubated with CD4^+^CD25^−^ T cells from a WT mouse infected two weeks earlier with influenza virus. These T-cell cultures were then stimulated *in vitro* with either influenza infected APCs or uninfected APCs. Influenza-specific proliferation was measured by [^3^H]-thymidine incorporation at day 5. As shown in Figures 7C and D, CD4^+^CD25^+^ Tregs isolated from IL-6^−/−^, but not WT mice infected 8 weeks (7D) but not 2 weeks (7C) previously with influenza virus, were able to suppress proliferation of WT influenza-specific CD4^+^ T cells. This data is consistent with our earlier observation that memory but not primary responses are impaired in influenza-infected IL-6^−/−^ mice and further supports the view that IL-6 has an inhibitory effect on the activity of virus-specific Tregs. We were unable to detect a greater increase in the number of Tregs (by Foxp3 staining) in the lymph nodes or spleens of influenza-infected IL-6^−/−^ compared to WT mice. The number of influenza-specific Treg may be very low thus detecting an increase in cell number in the overall Treg population as a result of the influenza virus infection might not be possible. No significant difference was observed in the ability of WT and IL-6^−/−^ CD4^+^CD25^+^ Tregs to suppress CD4^+^CD25^−^ T cell proliferation after stimulation with CD3-specific antibodies (Figure 7B) indicating that there is no inherent failure of Tregs to perform immunosuppressive functions in the IL-6−/− mice. Overall, these data clearly indicate that influenza-specific CD4^+^ T cells, from both WT and IL-6^−/−^ mice, can be suppressed by Tregs, and reveal a crucial role for IL-6 in inhibiting the activity of virus-specific Tregs.

## Discussion

In this study, we sought to address whether IL-6 plays a role in shaping the T cell response to influenza virus. This was considered a pertinent question since IL-6 has previously been shown to be an important survival factor for naïve T cells and to increase the frequency of antigen-specific CD4^+^ T cells primed *in vivo* [8],[11],[12]. To address this question we compared virus-specific CD4^+^ T cell responses in WT and IL-6^−/−^ mice approximately three months after infection with influenza virus. The results were striking. Whilst no difference was observed in the influenza-specific CD8^+^ T cell responses, CD4^+^ T cell responses were minimal in the absence of IL-6. The impairment in the CD4^+^ T cell responses was observed only during the memory phase of the infection and not the primary phase. Neutralising antibody responses were comparable in WT and IL-6^−/−^ mice though it is possible that over time, this antibody response as well as the CD8^+^ T cell response may wane more rapidly in the IL-6^−/−^ mice as a result of the defect in CD4^+^ T cell memory [13]. A previous publication by Kopf *et al*. reported that IL-6^−/−^ mice show a significant decrease in T-helper dependent CTL responses but not in those thought to be T-helper independent [14]. Similarly, the same study demonstrated that CD4^+^ T cell dependent IgG antibody production but not T cell-independent IgM responses were impaired in IL-6^−/−^ mice [14]. Thus, overall, it appears that IL-6 has a major effect on the adaptive immune system primarily through its influence on the activity of CD4^+^ T cells. The impact of the defect in CD4^+^ T cell memory on clearance of influenza virus was assessed in our study. When WT and IL-6^−/−^ mice infected with the H17 strain of influenza virus were subsequently rechallenged with the heterologous virus, PR8, we found a delay in virus clearance in IL-6^−/−^ mice compared to WT mice. Since antibodies capable of neutralizing the PR8 virus are not present in H17-memory mice, any protection against re-infection is likely to be conferred by cross-reactive T cells. Thus, it is likely that poorer control of virus in the IL-6−/− mice is attributable to impaired CD4^+^ T cell memory. Thus, whilst previous reports indicate no difference in virus replication in WT and IL-6^−/−^ mice infected for the first time with influenza virus [2],[3], protection against secondary infection may well be compromised when IL-6 is absent.

Although IL-6 has been shown to act as a survival factor for CD4^+^ T cells, we found no evidence of increased cell death amongst lymphocytes infiltrating the lungs of IL-6^−/−^ mice compared to WT mice either after the primary or secondary infection with influenza virus (data not shown). This observation implies that a defect in T cell survival does not account for the failure to detect an influenza-specific CD4^+^ T cell memory response in the IL-6^−/−^ mice. Recently, a series of reports have highlighted that IL-6 inhibits the activity of Tregs [9],[15]. We therefore hypothesized that in the absence of IL-6 Tregs may impinge upon the development of virus-specific memory T cells. Indeed, whilst influenza-specific CD4^+^ T cell memory responses were consistently low in IL-6^−/−^ mice, we found that simply depleting CD25^+^ cells (enriched for Tregs) from the population of splenic CD4^+^ T cells was enough to allow measurement of detectable anti-influenza virus responses in proliferation assays. An increase in the influenza-specific proliferative response was observed in a proportion of WT animals implying that Tregs do develop, albeit to a lesser extent, following infection of WT mice. To confirm that virus-specific Treg activity was more readily detectable in the absence of IL6, we stimulated co-cultures of CD4^+^CD25^−^ cells (obtained from influenza-infected WT mice) and CD4^+^CD25^+^ cells purified from either influenza-infected WT or IL-6^−/−^ mice with APCs infected with the virus. Suppression of the influenza-specific response was observed only in cultures containing CD4^+^CD25^+^ T cells purified from IL-6^−/−^ mice and not in cultures containing CD4^+^CD25^+^ T cells purified from WT mice. These data indicate that IL-6 normally limits the suppressive activity of Tregs either by restricting their expansion or their suppressive capacity. Influenza virus activates DCs through engagement with TLR 3, 7 and 8 [16],[17] , resulting in production of large amounts of IL-6 ([18] data not shown). Thus, influenza-infected DCs may represent at least one relevant source of IL-6 in the system under study here.

Our finding that IL-6 deficiency promotes the activity of antigen-specific Tregs parallels observations made in a murine model of asthma where blocking the mIL-6R with specific antibodies led to an increase in the number and suppressor activity of FoxP3 positive cells in the lungs of the experimental mice [15]. This report indicated that IL-6 activated Tregs via the mIL-6R whilst IL-6 responses in conventional effector T cells were elicited *via* its soluble receptor (sIL-6R), a process known as IL-6 trans-signaling (reviewed in [19]). However due to lack of reagents available to selectively manipulate IL-6 trans-signaling over the course of the 8–10 week viral infection, it is difficult to gauge whether a dual-mode of IL-6 signaling influences the outcome of the anti-influenza response. Our findings do however support the premise that the major target of IL-6 activity in our influenza model is the Treg population rather than conventional CD4^+^ T cells. Whilst the CD4^+^ memory T cell response is diminished in IL-6^−/−^ mice, due to the suppressive effect of Tregs, the memory CD8^+^ T cell response is unimpaired. This observation implies that CD4^+^ and CD8^+^ T cells differ in their susceptibility to suppression by Tregs. It is not clear why this should be the case since CD8^+^ T cells can be suppressed by Tregs under some circumstances [20]. Ultimately, a better understanding of how Tregs exert their suppressive effects *in vivo* will help clarify this issue.

Development of virus-specific Tregs in the absence of IL-6 may be explained by the recently described role of IL-6 in steering the commitment of naïve CD4^+^ T cells. Bettelli *et al*. demonstrated that IL-6 suppresses development of Tregs induced by TGFβ [10], a cytokine shown to facilitate differentiation and expansion of Tregs [21],[22]. With this in mind, it is possible that TGFβ is also produced during the immune response to influenza virus, which in the absence of IL-6 promotes expansion of Tregs. Bettelli *et al*. also showed that a combination of IL-6 and TGFβ directs the commitment of naïve T-cells towards an IL-17-secreting CD4^+^ T-helper (Th-17) population [10]. We saw no evidence of IL-17-secreting CD4^+^ T (Th-17) cells in the primary or secondary response to the influenza infection ruling out an essential role for these in development of virus-specific CD4^+^ T cell memory in WT mice.

Collectively, the data presented in this study point to a critical role for IL-6 in promoting memory CD4^+^ T cell responses. This may be due in part to the activities of IL-6 in promoting survival, proliferation and migration of antigen-specific T cells but as described in this study, a major pathway through which IL-6 promotes CD4^+^ T cell memory is through its inhibitory effect on virus-specific Tregs. The involvement of Tregs in infectious disease is curious. Many groups have studied Tregs in chronic infection and have, in some cases, attributed their involvement to a need for limiting pathogen-induced immunopathology [23],[24]). In the case of acute infection, the question that arises is why should Tregs be involved at all? Results of a recent study investigating the turnover of human Tregs imply that Tregs can arise from the antigen-specific memory T cell pool and are relatively short-lived compared to other T cells [25]. These findings led the authors to propose that antigen-specific Tregs facilitate resolution of an immune response without compromising the ability of the immune system to respond to a second challenge with the same antigen. These data imply that Tregs limit a primary T cell response without necessarily impinging on a memory response. Our findings imply that an absence of IL-6 favors conditions that enhance survival of Tregs allowing them to persist and inhibit the memory T cell response. The precise nature of these conditions remains to be defined. However it is conceivable that the cytokine milieu induced in response to antigenic challenge in the absence of IL-6 may influence the outcome of the response. In consideration of the question “why should Tregs play a role in acute infections?” - we would suggest that production of IL-6 by virus-infected cells serves two purposes – to promote priming of antigen-specific effector T cells, and to inhibit priming of antigen-specific Tregs thereby ensuring, within the time-frame of an acute infection, that antigen-specific Tregs have in fact, no opportunity to play a role.

Although the concerted activity of many arms of the immune system contribute to pathogen clearance, CD4^+^ T cells play a central role both in promoting and coordinating these activities and in facilitating long-term CD8^+^ T cell memory and B cell memory. Induction of a robust CD4^+^ T cell response is therefore likely to be critical to the success of any vaccine strategy. The results of this study imply that co-administration of IL-6 with an anti-influenza virus vaccine will promote development of protective immunity through optimal induction of a virus-specific memory CD4^+^ T cell response.

## Materials and Methods

### Influenza virus

Recombinant Influenza A virus strain E61-13-H17 (H17, H3N2) and Influenza A virus strain A-PR8-34 (PR8, H1N1), were obtained from the National Institute for Medical Research (London, UK) [26], Both H17 and PR8 were amplified in embryonated chicken eggs as described previously and haemagglutination assays conducted to determine the viral titre [27].

### Infection of mice with Influenza virus

Experiments were conducted on 6–8 week old, female, C57/BL/6 wild-type (WT) and IL-6 deficient (IL-6^−/−^) mice, bred in-house at Cardiff University. Mice were housed in individually ventilated cages and allowed access to standard mouse chow and water ad libitum. Mice were infected intra-nasally (i.n.) with either 20 HAU of H17 or PR8 in 20 µl of sterile PBS, as indicated in individual experiments. All experimental procedures conducted were in compliance with the UK Home Office.

### Analysis of serum cytokines

The presence of IL-6, IL-10, MCP-1, IFNγ, TNFα, and IL-12 was assessed in serum derived from naïve, uninfected mice or from mice 3 and 8 days post infection with H17. An Inflammatory Cytometric Bead Array Kit (BD Biosciences, USA) was used according to the manufacturers' guidelines and the presence of each cytokine was analysed by flow cytometry (FACS-CALIBUR®; Becton Dickinson, CA, USA).

### Cytotoxic lymphocyte (CTL) assay

4×10^6^ splenocytes derived from WT and IL6^−/−^ mice at either 2 or 8 weeks post-primary infection with H17 were stimulated with 1×10^6^ irradiated APCs loaded with 100 µl NP68 peptide (10^−6^ M) and incubated at 37°C. After 2 days in culture, IL-2 (10 units/ml) was added and the cells cultured for an additional 3 days. Cultures were then diluted 1∶3 and incubated for 4–5 hours in the presence of ^51^Cr-labelled, NP68 peptide- or irrelevant peptide-loaded B16 cells (1×10^4^ cells/well) at 37°C. To generate maximal lysis, 5% Triton ×100 was added to the cultures, whilst culture media alone was added as an indication of minimal lysis. Gamma emission was measured as a readout of ^51^Cr-release from the cells. Influenza specific CTL activity was measured as described previously.

### Flow cytometry and FACS analysis

Anti-CD4-FITC and anti-CD4-alexa fluor 647 were purchased from Caltag Laboratories (Burlingame, CA, USA). Anti-CD8-PerCP-Cy5.5, anti-CD4-PerCP-Cy5.5, anti-IL-6Receptor-PE, anti-IFNγ-APC, anti-CD25-FITC, anti-TNFα-alexa fluor 488, anti-CD16/CD32-Fc Block, streptavidin-PerCP-Cy5.5 were purchased from BD Pharmingen (San Diego, CA, USA). Anti-IL-6Receptor-purified was purchased from Biolegend (San Diego, CA, USA). Chicken-anti-rat-alexa fluor 647 was purchased from Invitrogen Molecular Probes (Paisley, UK). Anti-IL-6Receptor-biotinylated was purchased from R & D Systems (Minneapolis, USA). The PE-labelled NP tetramer (D^b^-ASNENMDAM [28]) and control GP tetramer (Db-KAVYNFATC) used in this study was generated in-house using previously described methods [29]. Cells stained with directly conjugated antibodies were incubated with 1–2 µg/ml of antibody for 30 minutes at 4°C prior to washing and resuspending in FACS buffer (PBS, 2% FCS, 2 mM EDTA). Unconjugated and biotinylated antibodies required secondary staining with 1 µg/ml of chicken anti-rat alexa fluor 647 and streptavidin-PerCP-Cy5.5 respectively, prior to washing and resuspension in FACS buffer. Cells were fixed with FACS Fix buffer (PBS, 2% FCS, 2 mM EDTA, 2% Formalin) prior to analysis.

In all cases the cells were analysed by flow cytometry (FACS-CALIBUR®; Becton Dickinson, CA, USA) and the data analysed using FlowJo Software (Ashland, OR, USA).

### Intracellular TNFα staining

Single cell suspensions were prepared from spleens of mice 8 weeks after primary H17 infection. Cells were incubated at 37°C for 5 hours in the presence of CD28 (1 µg/ml), BrefeldinA (1 µg/ml) and 10 HAU of H17 or 10^4^ PFU of recombinant vaccinia virus as an irrelevant virus control. Following incubation the cells were washed with FACS buffer and stained for intracellular TNFα using a Cytofix/Cytoperm Kit (BD Pharmingen, San Diego, CA, USA) according to the manufacturers instructions, prior to resuspension in FACS buffer and 100000 lymphocytes were acquired on a FACSCalibur flow cytometer. Total numbers of TNFα^+^ CD4^+^ T cells were calculated based on percentage of TNFα^+^ cells analyzed by flow cytometry.

### CFSE labelling and intracellular IFNγ staining

A single cell-suspension of splenocytes was prepared from the spleens of mice at day 4 post-rechallenge with PR8 virus. Splenocytes were labelled with 0.5 mM CFSE (Molecular Probes) and 5×10^5^cells/well were stimulated with 10 HAU of H17 and CD28 (1 µg/ml). Cells were incubated for 6 days at 37°C. For intracellular IFN-γ detection, influenza pulsed dendritic cells (DCs) were generated from splenic DCs derived from naïve WT mice, isolated by low density gradient centrifugation as previously described [30] and incubated overnight with 400 HAU of H17. Following 6 days incubation proliferating cells were washed with culture media (RPMI+10% FCS) and incubated in the presence of CD28 (1 µg/ml), BrefeldinA (1 µg/ml) and influenza-pulsed DCs for 5 hours at 37°C. Intracellular staining for the presence of IFNγ was performed as outlined previously.

### Determination of H17 neutralizing antibody titres

Serum from both WT and IL-6^−/−^ mice was removed at 6–8 weeks post primary infection with H17 and from uninfected, naïve WT mice. MDCK cells at 50% confluence were plated into flat-bottomed 96 well plates and cultured in the presence of 10 HAU of H17 and serum at a starting concentration of 1∶10 and three-fold dilutions thereafter. The cells were cultured for 3 days at 37°C prior to the assessment of cell viability using alamar blue (Biosource International, Carmillo, CA, USA). The viability of the cells was calculated according to the manufacturers' guidelines. Data is expressed as the antibody concentration at which the infectivity of 10 HAU of H17 virus was inhibited by 50%.

### Determination of relative virus load

Lungs from mice infected with H17 and rechallenged with PR8 were extracted at day 4 post-secondary infection and homogenised in 1ml of serum free media. MDCK cells at 50% confluence were plated into flat-bottomed 96 well plates and cultured with three-fold dilutions of lung homogenate. The cells were cultured for 3 days at 37°C prior to the assessment of cell viability using alamar blue (Biosource international, Carmillo, CA, USA). The viability of the MDCK cells cultured with a standard PR8 virus preparation (positive control), no virus (negative control) and different dilutions of lung homogenate was assessed.

### Influenza virus-specific CD4^+^ T cell proliferation

Splenic CD4^+^ T cells derived from WT and IL-6^−/−^ mice at 6–8 weeks post-primary H17 infection were purified by positive MACS microbead selection (Miltenyi Biotec, Bergisch-Gladbach, Germany) according to the manufacturers' instructions. Splenocytes derived from a naïve, WT mouse were used as a source of APCs and were infected with 100 HAU of inactivated H17 for 1–2 hours at 37°C prior to irradiation at 2400cGy. Non-influenza infected, irradiated splenocytes were used as a negative control. 1×10^5^ CD4^+^ ‘effector’ cells were cultured in the presence of 6×10^5^ APCs in 96 well round bottom plates. Cells were incubated for 6 days at 37°C, with [^3^H]-thymindine added for the final 18 hours of the study. Cell proliferation was assessed by [^3^H]-thymidine incorporation.

### Suppression assays

CD4^+^ CD25^+^ T cells were purified from splenocytes, derived from WT and IL-6^−/−^ mice at 8 weeks post-primary H17 infection, using a CD4^+^ CD25^+^ Regulatory T Cell Isolation Kit (Miltenyi Biotec, Bergisch Gladbach, Germany) according to the manufacturer's instructions. 1×10^5^ CD4^+^ ‘effector’ cells from a WT mouse at 2 weeks post-primary H17 infection, were isolated as described previously, and incubated with the CD4^+^ CD25^+^ T cells at a ratio of 1∶1 in 96 well plates. The CD4^+^ ‘effector’ cells were stimulated to proliferate by the addition of either 6×10^5^ irradiated splenic APCs, infected with 100 HAU of inactivated H17 or treated with 1 µg/ml of anti-CD3 mAb to the cells. Cells were incubated for 6 days at 37°C, with [^3^H]-thymindine added for the final 18 hours of the study. Cell proliferation was assessed by [^3^H]-thymidine incorporation.

### Statistical testing

All statistical differences determined in this study used the paired means Students T test. P values of ≤0.05 were considered significant (*), with values of ≤0.01 considered highly significant (**).
